# A cross-sectional study on the moderating effect of self-efficacy on the relationship between sociodemographic variables and nutrition literacy among older adults in rural areas of North Sichuan

**DOI:** 10.3389/fnut.2023.1335008

**Published:** 2024-01-08

**Authors:** Shasha Liu, Xiaomei Fan, Li Jiang, Tao Liu

**Affiliations:** ^1^Department of Nursing, Jinzhou Medical University, Jinzhou, China; ^2^Chengdu BOE Hospital, Chengdu, China; ^3^First Affiliated Hospital of Chengdu Medical College, Chengdu, China

**Keywords:** elderly, demographic variables, nutrition literacy, self-efficacy, moderating effect

## Abstract

**Objective:**

The purpose of this study is to examine the moderating role of self-efficacy among rural elderly individuals in northern Sichuan Province in the relationship between certain sociodemographic variables and nutritional literacy.

**Methods:**

Convenience sampling was used to select 264 elderly individuals aged 60 and above from rural communities in Cangxi County, Guangyuan City, Yilong County, Nanchong City, and Bazhou District, Bazhong City, Sichuan Province. A self-designed questionnaire, including sociodemographic variables, the General Self-Efficacy Scale (GSES), and the Nutrition Literacy Questionnaire for the Elderly (NLQ-E), was administered through face-to-face interviews using a paper-based version. The relationships between sociodemographic variables, self-efficacy, and nutritional literacy in the elderly were analyzed using SPSS 26.0 and the Process plugin to examine the relationships between variables and to test for moderation effects.

**Results:**

(1) There were significant differences in nutrition literacy scores among elderly people of different ages, genders, marital statuses, educational levels, personal monthly living expenses, dental conditions, and number of chronic diseases (*p* < 0.05). (2) When elderly individuals have lower self-efficacy, their nutritional literacy is lower as they become older, and they have poorer nutritional literacy with a higher number of chronic diseases.

**Conclusion:**

General population demographic data has a significant impact on the nutritional literacy level of elderly people in rural areas of northern Sichuan. Self-efficacy plays a moderating role in the relationship between age and nutritional literacy, as well as the relationship between the number of chronic diseases and nutritional literacy.

## 1 Introduction

Health is the cornerstone of human development, and nutrition is the cornerstone of maintaining health. Nutrition refers to the process by which the human body ingests, digests, and absorbs various nutrients from the outside world to maintain life activities and regulate the immune system to change the course of disease (1). With age, the body’s metabolism slows down, and the physiological functions of various systems decline, making the elderly more prone to poor nutrition and overnutrition. According to statistics, the risk of malnutrition in Chinese elderly is as high as 48% (2). As of 2019, the population aged 65 and above in China reached 176.03 million, accounting for12.6% of the total population; the population aged 80 and above reached 26 million; by 2050, the elderly population aged 65 and above will reach 365 million, accounting for 26.1% of the total population (3). Therefore, if effective measures are not implemented, the current situation 30 years later will be that about 1 in 12 people will be malnourished elderly. And due to the differences in conditions between rural and urban areas in China (4), this situation will be even more worrying. Therefore, reducing the risk of malnutrition and improving the current situation of malnutrition is essential for personal and social harmony and development.

Nutrition literacy is the ability of individuals to understand nutritional knowledge, their own nutritional status, and guide themselves in selecting foods to obtain nutrition, which is a part of health literacy (5, 6). At the same time, healthcare professionals also regard improving the nutrition literacy of chronic disease patients as an important part of their health guidance, and these measures play an important role in the prevention and treatment of nutrition and metabolism-related diseases such as hypertension, diabetes, obesity, and cancer (7–10). Therefore, from the concept and application, nutrition literacy is the upstream theory of nutrition, that is, an individual’s level of nutrition literacy directly affects their nutritional status.

The differences in physiological and psychological status, living environment, cultural level, and economic level among the elderly lead to differences in their nutrition literacy (11). Aihara used a nutrition literacy scale suitable for the Japanese elderly based on the Japanese Dietary Guidelines to assess the elderly’s understanding of nutrition-related knowledge. He found that there was a significant difference in nutrition literacy between genders, and lower cultural and economic status were related to limited nutrition literacy among elderly women (12). Prof. Zhang from the School of Public Health, Peking University, developed a nutrition literacy questionnaire suitable for Chinese elderly, and pointed out that elderly people with lower age, higher BMI, and higher cultural level have significantly higher nutrition literacy (2). In addition, the nutrition literacy of the elderly is also related to their oral health. A cross-sectional study from Finland found that elderly people who wear full dentures tend to choose unhealthy foods, leading to malnutrition (13).

Improving nutrition literacy is an important foundation for individuals to maintain a healthy state and an important means for the country to promote healthy aging. With the development of social psychology, the impact of positive psychological resources on health-promoting behaviors has received widespread attention, and more and more research has begun to pay attention to the relationship between elderly people’s psychological changes and their health status (14, 15). In this field, research on the relationship between self-efficacy and health-promoting behaviors among the elderly involves more (16). This relationship refers to whether the elderly can adopt adaptive behaviors to reduce the harm of events to their own health when facing stress or challenges in the environment, and self-efficacy is a psychological cognition related to this behavior (17–19). A survey on adult health literacy in Germany showed that demographic factors have an important impact on their health literacy level. But the effect size of these factors changed after adding self-efficacy to the regression model (20). The phenomenon of change occurring due to the addition of self-efficacy aligns with Bandura’s theory of self-efficacy (21). Therefore, in formulating research on improving nutritional literacy in the elderly, it would be highly valuable to explore the relationship between sociodemographic factors and nutritional literacy, while incorporating Bandura’s theory of self-efficacy.

Currently, nutrition literacy related research in the Chinese population mainly focuses on the development of scales (22). While the relationship between demographic data and nutrition literacy, as well as the underlying mechanisms, are not clearly understood. Therefore, this study aims to investigate the current status of nutrition literacy among elderly people in rural areas and promote local healthy aging. Based on the research objects and significance of the study, we proposed two hypotheses:

*Hypothesis 1*: The nutritional literacy of the elderly varies with differences in certain demographic variables.

*Hypothesis 2*: According to the theory of self-efficacy, this study assumes that general self-efficacy has a moderating effect on the relationship between certain demographic factors and nutrition literacy.

## 2 Materials and methods

**Figure 1:**
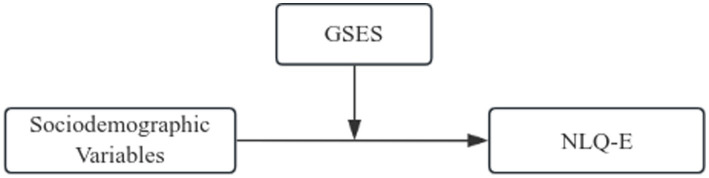
Moderation effect pathway.

The present study is based on the promotion effect of positive psychological resources on health behaviors. It explores the moderating effect of self-efficacy in rural elderly individuals on the relationship between sociodemographic variables and nutritional literacy. General sociodemographic data are used as independent variables, general self-efficacy is the moderating variable, and nutritional literacy is the dependent variable to examine the relationship among the three factors. The path relationships are presented in Figure 1.

### 2.1 Study population

This study used convenience sampling to select three rural communities in Guangyuan, Nanchong, and Bazhong cities in Sichuan Province, China. The selected participants were aged 60 years or older and had lived in the local rural areas for at least 1 year. The research team, accompanied by local community workers, we asked elderly individuals who voluntarily answered the questions and had normal cognitive function (not diagnosed with dementia or any mental illness by a doctor). Since the ultimate goal of this study was to investigate the nutrition self-management status of elderly people, elderly people who were completely unable to take care of themselves were excluded during the questionnaire survey stage. According to the Kendall sample calculation method, the sample size should be 5–10 times the maximum number of items on the scale. Taking into account a 20% sample loss, the minimum sample size required for this study is 240. The final number of questionnaires collected that met the inclusion criteria is 264.

### 2.2 Selection of demographic variables

The survey questionnaire for this project included seven demographic variables, including five basic characteristics: age, gender, marital status, education level, and personal monthly living expenses. According to a systematic review published by the Department of Periodontology of the Federal University Rio Grande do Sul, elderly people with more teeth and functional teeth units have better nutritional status (23). Therefore, this study also added the dental status of elderly people in the local area as a basic characteristic in the questionnaire. It has been reported that elderly people with nutrition-related chronic diseases tend to a Mediterranean-style diet, while those who choose a high-calorie, high-fat, and high-salt diet have little knowledge about diet and disease (24). Therefore, the number of chronic diseases related to health in elderly people was also added to the basic information questionnaire in this study.

### 2.3 General self-efficacy scale

Self-efficacy is a core concept in Bandura’s social cognitive theory. According to Bandura’s theory, people with different levels of self-efficacy feel, think, and act differently (25). People with high self-efficacy tend to choose more challenging tasks, work harder once they start, persist longer, and quickly recover from setbacks. The General Self-Efficacy Scale (GSES) was revised by (26) and is a quantitative measure of an individual’s psychological activity and self-confidence when facing setbacks or difficulties. The initial version consisted of 20 items, but it was later revised in 1997 to include 10 items and 1 dimension. The score of the GSES is obtained by summing the scores of the 10 items. The scores are rated on a 4-point Likert scale, where “1” means “completely incorrect,” “2” means ewhat correct,” “3” means “mostly correct,” and “4” means “completely correct.” The scale has been translated into 25 languages and is widely used. The Chinese version of the General Self-Efficacy Scale was used in this study, and its Cronbach’s α coefficient was 0.89 (27).

### 2.4 Nutrition literacy questionnaire for the Chinese elderly

There are many different versions of nutrition literacy scales published both domestically and internationally. The Nutrition Literacy Assessment Instrument (NLit) was designed by Gibbs (28). The scale covers all stages of obtaining nutrition and has been well validated in various populations. The Iranian version of FNLIT and the Turkish version of ANLS are nutrition and food literacy evaluation scales (29, 30). The questionnaire used in this study was the Chinese Elderly Nutrition Literacy Survey Questionnaire developed by Professor Zhang from the School of Public Health at Peking University (2). The questionnaire has 20 core items in three dimensions: the first 6 items evaluate the elderly’s nutritional knowledge, the next 9 items evaluate the elderly’s knowledge of healthy lifestyles and dietary behaviors, and the last 5 items evaluate the elderly’s knowledge of promoting nutrition-related skills. The scores are rated on a 5-point Likert scale, where “1” means “strongly disagree,” “2” means “disagree,” “3” means “uncertain,” “4” means “agree,” and “5” means “strongly agree.” The total score ranges from 20 to 100, with a higher score indicating higher nutrition literacy, The Cronbach’s α coefficient for the scale used in this study is 0.926.

### 2.5 Statistical analysis

Before conducting statistical analysis, the first author reviewed all the included questionnaires again, and the data were entered independently by the first and second authors. SPSS26.0 was used for statistical. Since the sample data did not meet the normal distribution, median was used to describe the general information. The K-W test was used for intergroup comparison of nutrition literacy scores. The relationship between elderly self-efficacy and nutrition literacy was analyzed using Spearman’s correlation. The moderating effect analysis was performed using PROCESS plug-in (by conducting 5,000 simulations using self-sampling, a 95% confidence interval is obtained), with chronic disease number and age as independent variables, self-efficacy score as moderating variable, and nutrition literacy score as dependent variable. Based on the results, a Johnson-Neyman plot was created (31). To ensure the accuracy of data analysis, data standardization, dummy variables, and multicollinearity diagnosis were performed before regression analysis. Considering that the questionnaires used in this study are widely used and established scales, no common method bias test was performed.

## 3 Results

### 3.1 Nutritional literacy score for the elderly based on demographic data

In this study, significant differences were found in the nutritional literacy scores of elderly individuals across various demographic variables, including age, gender, marital status, education level, monthly living expenses, tooth condition, and chronic diseases (*p* < 0.05). Please refer to Table 1 for detailed results. Additionally, comparative analysis was conducted within each demographic variable for all samples that exceeded the minimum required sample size. Further information can be found in the supplementary materials.

**Table 1:** Comparison of nutritional literacy scores among older adults based on demographic data (*n* = 264).

Characteristics		*n* (%)	NLQ-E(median)	*p*
Age	60–69	115 (43.6%)	80 (77, 97)	<0.001
	70–79	109 (41.3%)	77 (66, 80)	
	≥80	40 (15.2%)	62 (40, 72)	
Gender	Male	131 (49.6%)	80 (76, 97)	<0.001
	Female	133 (50.4%)	73 (63, 78)	
Marriage status	Never married	1 (0.4%)	79	<0.05
	Married	200 (75.8%)	78 (71, 88)	
	Divorced/Widowhood	63 (23.9%)	72 (61, 80)	
Education level	Illiteracy	97 (36.7%)	66 (51, 77)	<0.001
	Primary school diploma	94 (35.6%)	79 (73, 82)	
	Junior high school diploma	70 (26.5%)	88 (80, 98)	
	High school diploma or above	3 (1.1%)	100 (88,100)	
living expenses CNY/month	≤500	101 (38.3%)	68 (53, 78)	<0.001
	501–999	112 (42.4%)	78 (73, 87)	
	1,000–1999	46 (17.4%)	97 (81, 100)	
	≥2000	5 (1.9%)	91 (77, 100)	
Tooth condition	False tooth	100 (37.9%)	73 (66, 78)	<0.001
	Damaged teeth without dentures	90 (34.1%)	78 (66, 80)	
	Intact teeth	71 (26.9%)	84 (77, 97)	
	Toothless	3 (1.1%)	63 (40, 68)	
Chronic diseases	0	117 (44.3%)	80 (77, 97)	<0.001
	1	113 (42.8%)	72 (66, 80)	
	2	32 (12.1%)	71 (52, 80)	
	≥3	2 (0.8%)	41 (40, 42)	

### 3.2 Correlation analysis between self-efficacy and nutrient literacy scores among rural elderly

As shown in Table 2, based on Spearman correlation, the correlation coefficients between self-efficacy scores and nutrient literacy scores in each dimension ranged from 0.754 to 0.779 among the 264 elderly participants in this study. The correlation coefficient between self-efficacy scores and the total score of the nutrient literacy questionnaire was 0.800, indicating a strong positive correlation between self-efficacy and nutrient literacy among the population surveyed in this study.

**Table 2:** Spearman correlation coefficients between self-efficacy and nutritional literacy dimensions.

Variables	Healthy lifestyle and dietary behavior	Healthy lifestyle and dietary behavior	Skill	Total
GSES	0.779^**^	0.764^**^	0.754^**^	0.800^**^

*p < 0.05, **p < 0.01.

### 3.3 Self-efficacy moderates the effects of sociodemographic variables on nutritional literacy among elderly individuals in rural areas

Before performing the moderation analysis, this study conducted a collinearity diagnosis on the included variables. The test results showed that the VIF < 5. The results are shown in Table 3, indicating that when the elderly residents’ self-efficacy score is used as the moderating variable, there is a significant effect (*p* < 0.05) on the impact of age and the number of chronic diseases on their nutritional status. To demonstrate the moderating effect of self-efficacy among elderly residents, this study will quantify the impact of age and the number of chronic diseases on nutritional status under different self-efficacy scores, and present the results in two Johnson-Neyman graphs.

**Table 3:** Results of regression analysis on regulatory effectiveness.

		β	se	*t*	*p*	95%CI	
Model 1	Constant	0.052	0.041	1.267	0.206	−0.029	0.133
	Age	−0.130	0.043	−3.030	0.003	−0.214	−0.045
	GSES	0.691	0.043	16.108	0.000	0.607	0.776
	Int_1	0.107	0.039	2.738	0.007	0.030	0.184
	*R* ^2^	0.651			0.000		
	△*R*^2^	0.010			0.007		
Model 2	Constant	0.044	0.041	1.070	0.286	−0.037	0.125
	Chronic diseases	−0.099	0.042	−2.367	0.019	−0.182	−0.017
	GSES	0.726	0.042	17.231	0.000	0.643	0.809
	Int_1	0.097	0.040	2.454	0.015	0.019	0.175
	*R* ^2^	0.641			0.000		
	△*R*^2^	0.008			0.015		

#### 3.3.1 Analysis of the impact of self-efficacy regulation age on nutritional literacy in older adults

**Figure 2:**
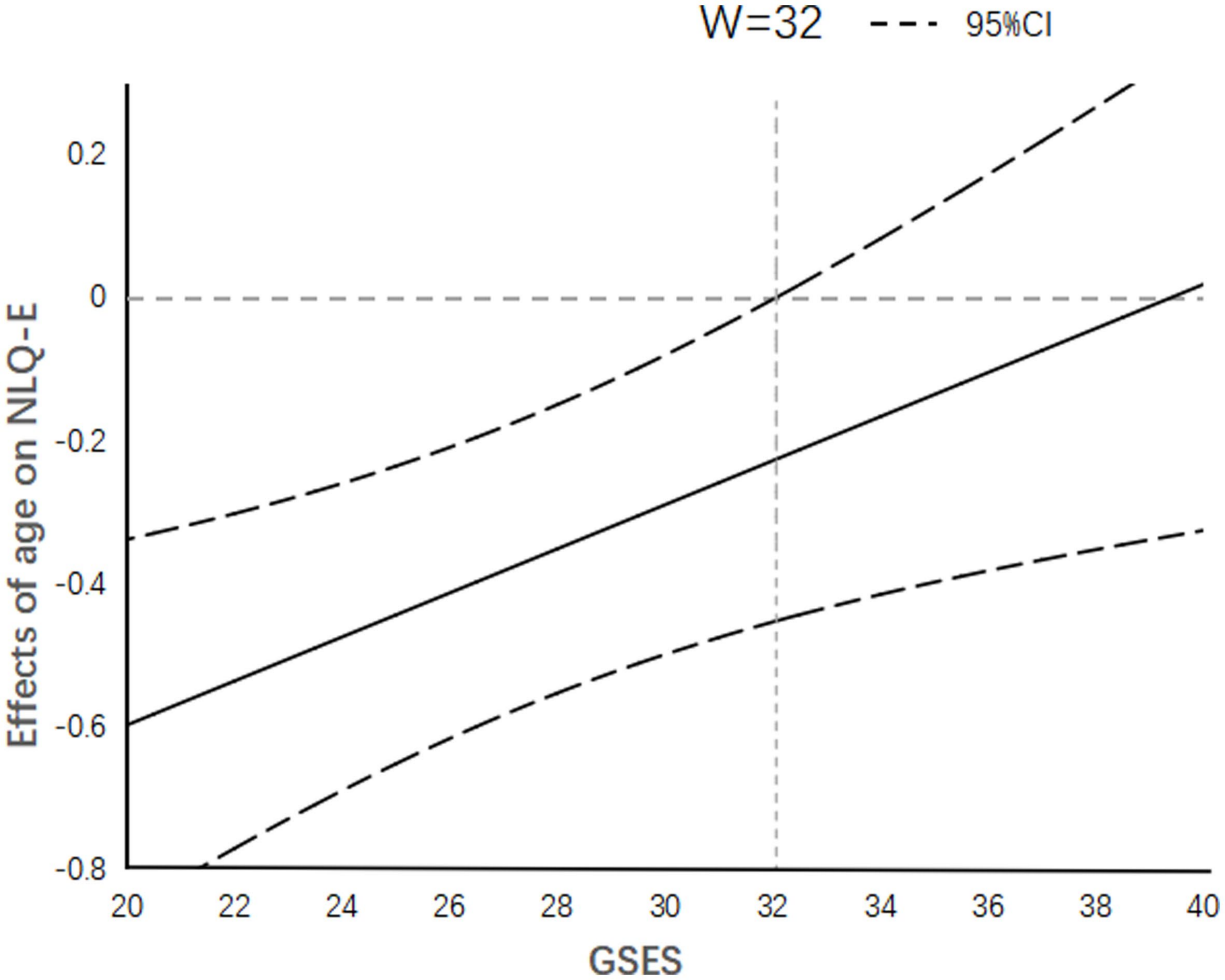
Age has a significant negative effect on nutritional literacy when self-efficacy score is below 32.

As shown in Figure 2, when the self-efficacy score of elderly people is below 32 points, the effect of age on nutritional status is below 0, indicating a significant negative effect of age on nutritional status. While when the self-efficacy score is above 32 points (The confidence interval of the effect size includes 0) indicating that the effect size in this stage is meaningless. According to relevant literature, elderly people with self-efficacy below 30 points generally belong to the group of poor self-efficacy (32). However, this study found a critical score of 32 points. Therefore, the study results indicate that elderly people with lower self-efficacy generally have lower nutritional status scores with increasing age, implying that this group of elderly people has lower ability to obtain nutrition.

#### 3.3.2 The effect of self-efficacy in regulating the number of chronic diseases on the nutritional literacy score in elderly individuals was analyzed

**Figure 3:**
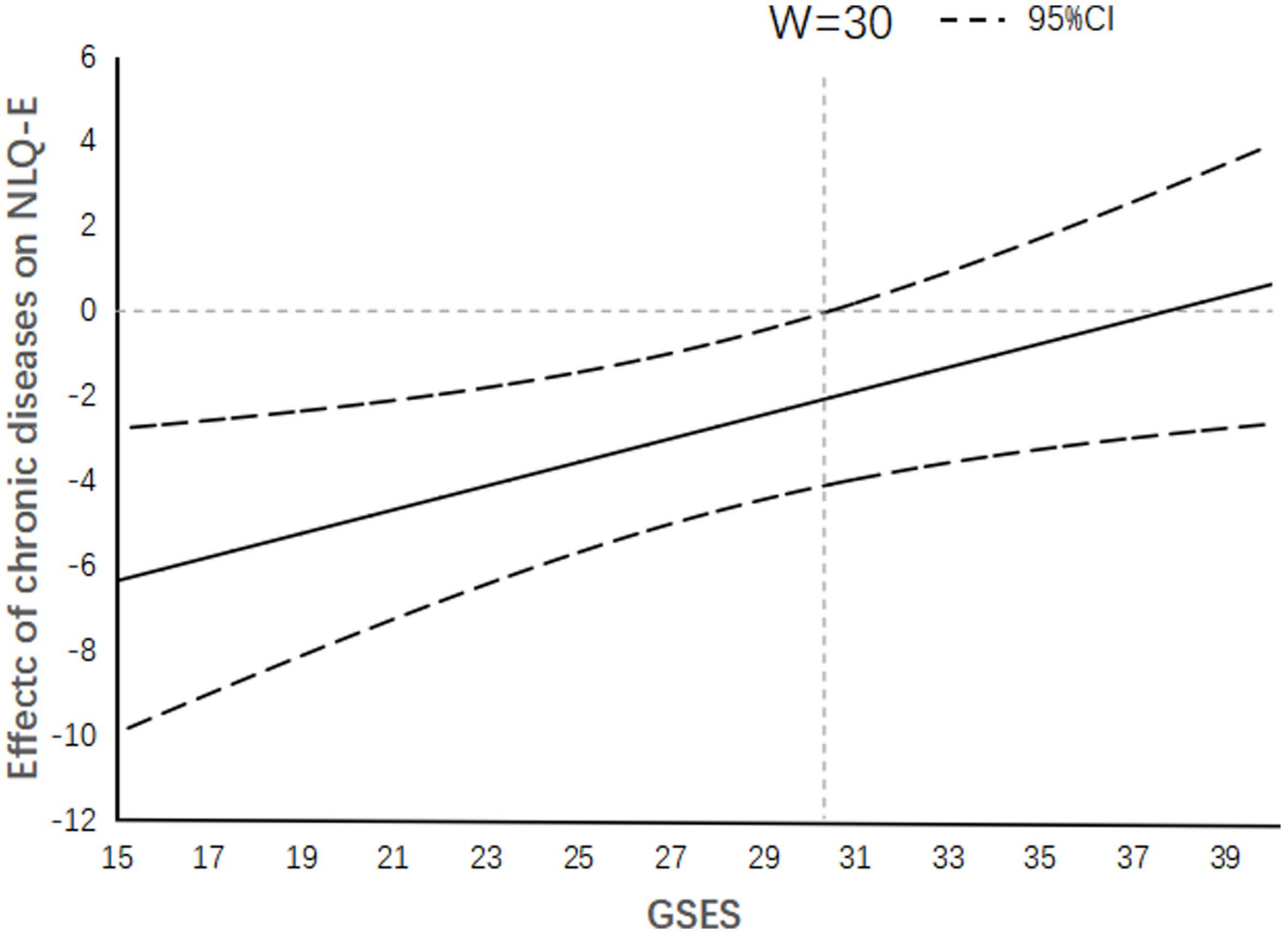
When the self-efficacy score is less than 30 points, the number of chronic diseases has a significant negative effect on nutritional literacy.

As shown in Figure 3, the confidence intervals for the impact of the number of chronic diseases on nutritional status among older adults do not include zero when the self-efficacy score is below 30, and they are all below 0. This indicates that the effect of having fewer chronic diseases on higher nutritional status is significant only among older adults with self-efficacy scores below 30. However, among older adults with higher self-efficacy scores, the impact of the number of chronic diseases on nutritional status loses theoretical significance (The confidence interval of the effect size includes 0).

## 4 Discussion

### 4.1 Focus on the baseline characteristics of the elderly population and improve the nutritional literacy of key populations

The results of this study indicate that age is an important influencing factor in evaluating the nutritional literacy of the elderly. As age increases, the physiological indicators of the elderly decline, and their bodily functions decrease. Which may lead to the need for more nutritional substances but also require more energy to obtain (33). However, they often lower their nutritional requirements due to lack of energy, forcing them to have insufficient ability to obtain nutrients, resulting in a significant decrease in nutritional literacy with increasing age. A study on the age distribution of nutritional and health knowledge among Chinese adults (34) conducted a multicenter large-sample survey. The results showed that as age increases, nutritional knowledge among Chinese adults declines significantly. These elderly individuals have not obtained more information about nutrition from healthcare providers to compensate for this knowledge gap (35). Therefore, this study explained the relationship between age and nutritional literacy from a more specific dimension.

However, there is a lack of multicenter large-sample studies on the impact of gender on the nutritional literacy of the elderly. A study in Taiwan, China, on the impact of gender on food choices among the study population (11) supports the view of this article that the nutritional literacy score of elderly males is higher than that of elderly females. That is, elderly males show significantly higher initiative in adopting health-related dietary behaviors than females. Another cross-sectional survey with a larger sample size from Japan showed that nutritional literacy shows gender differences in different dimensions of dietary quality and food choice value (36). Therefore, further research may be needed on the analysis of the impact of gender on nutritional literacy.

The results of this study showed that the nutritional status of married elderly people was significantly higher than that of divorced or widowed individuals. However, there is currently a lack of literature to support or refute the impact of marital status on the nutritional status of elderly people. Married elderly people may be more willing to invest more energy in obtaining better nutrition due to emotional support from their spouses and daily care. Previous literature has reported that elderly people living alone suffer more severe health impacts due to a lack of social support (37).

The nutritional status of the elderly people surveyed in this study showed significant differences in the cultural background level. A large-scale survey conducted in China showed that elderly people in low-culture rural areas had limited nutritional status due to their lack of knowledge about diet (38). Using the theory of knowledge, attitudes, and behavior, it can be inferred that a person’s cultural level has a direct impact on their nutritional behavior (39). Therefore, the conclusion drawn in this study also confirms the reliability of this theoretical model. Another study on the nutritional status of elderly people in Spain, although using different questionnaires (40), also drew similar conclusions to this study: the nutritional status of elderly people with lower cultural education is significantly lower than that of college-educated elderly people, indicating that the nutritional status of elderly people increases with higher cultural education.

The economic source of rural elderly people surveyed in this study is mainly from farming (41), and the basic part of their daily diet is self-sufficient. A large part of their personal monthly living expenses is used to pay for additional nutritional supplements. Therefore, this study selected the personal monthly living expenses of elderly people as an economic indicator to measure their nutritional support expenditure. According to the results of this study, it is speculated that under a relatively low socioeconomic status, the reason why personal monthly living expenses have a significant impact on their nutritional status may be due to the differences in their purchasing power resulting in significant differences in the nutritional substances they choose. Currently, research has shown that guiding low-income people to choose nutritious and affordable foods can reduce the difference in nutritional status (42). The promotion of such intervention measures may have significant benefits in improving the nutritional status of low-income elderly people.

In this study, the nutritional status scores of elderly people with intact teeth and those with damaged teeth differed significantly, but the difference in whether dentures were worn by elderly people with damaged teeth was not clear. The convincing reason for this conclusion comes from the fact that the oral environment, especially the condition of the teeth, has a significant impact on the food choices of most elderly people. The limited medical level in rural areas of China means that elderly people cannot choose more scientific treatment options after their teeth are damaged, and most elderly people’s dentures cannot effectively chew food (43). The number of edentulous elderly people included in this study was limited, so effective statistical analysis was not carried out. The wearing of dentures was not further subdivided into more detailed categories, so it was not possible to make a more accurate judgment. However, research has shown that patients wearing full dentures tend to choose unhealthy foods (13), leading to a higher risk of malnutrition, which needs further exploration in future studies.

Elderly people with chronic diseases, especially those related to nutrition, often have dietary habits and lifestyles that are closely related to the occurrence and development of chronic diseases (44). The results of this survey show that the nutritional literacy scores of elderly people with chronic diseases are significantly lower than those of healthy elderly people, indicating a certain relationship between chronic diseases and nutritional literacy. An experimental study in the United States provided food resources and cooking skills for hypertensive patients, which can improve their self-management level and increase their nutritional literacy (45). Based on this idea, promoting healthy lifestyles, especially dietary-related skills, to the public may reduce the incidence of chronic diseases by improving nutritional literacy. Finally, this study also showed that there was no significant difference in the effect of the number of chronic diseases on the nutritional literacy of the elderly. There is currently no relevant evidence to prove this, which may be due to the insufficient sample size collected in this study and further research is needed to verify this.

### 4.2 Focus on the self-efficacy of the elderly population and assist in screening for nutritional literacy

The nutritional literacy of the elderly population varies not only due to their different baseline characteristics but also as a result of their psychological state (11). Correlation analysis in research results has shown a strong positive correlation between self-efficacy and nutritional literacy among the elderly, and multiple studies abroad have also demonstrated a strong correlation between self-efficacy and nutritional literacy (46, 47). Therefore, self-efficacy, as a positive psychological resource, can be used to assess the confidence of the elderly in their ability to obtain nutrition. Relevant studies have already confirmed that the self-efficacy of elderly people in rural China is relatively low and significantly different from that of elderly people in urban areas (48). So focusing on the self-efficacy of rural elderly populations will be an important measure to improve their nutritional literacy.

Self-efficacy theory is an important component of psychologist Bandura’s social learning theory, which emphasizes the mediating role of self-efficacy in behavior (21). Based on this theory, the present research results show that self-efficacy, as a moderating variable, affects the predictive effect of age and the number of chronic diseases on the nutritional literacy of the elderly. A survey on adult health literacy in Germany obtained results similar to those of this study (20), showing that the effect size of sociodemographic variables on nutritional literacy changed after self-efficacy was added to the regression model. Therefore, these studies suggest that when considering the impact of baseline characteristics on the nutritional literacy of the elderly, the position and role of self-efficacy in the model should be considered. Based on the Johnson-Neyman plot, the present research results show that when the self-efficacy of the surveyed elderly population is poor, age has a negative effect on nutritional literacy, that is, the nutritional literacy of the elderly decreases with age. Therefore, by using the self-efficacy score of rural elderly populations as a basis and dividing them into low self-efficacy score groups and other groups, the overall literacy level of rural elderly populations can be improved by implementing interventions to improve their nutritional literacy, especially for older elderly people. Similarly, it can be seen from the Johnson-Neyman plot that when self-efficacy is low, the number of chronic diseases also has a negative effect on nutritional literacy. Therefore, elderly people with chronic diseases in the low self-efficacy group can be screened out for targeted nutrition education and interventions (49). Therefore, this study suggests that when assessing the nutritional literacy of rural elderly populations, a general self-efficacy assessment scale can be used for auxiliary screening. On the one hand, self-efficacy itself has a positive predictive effect on nutritional literacy. And on the other hand, targeted interventions for elderly people with low self-efficacy scores who are older or have chronic diseases can significantly improve the overall nutritional literacy level. Additionally, the general self-efficacy assessment scale is simple and easy to evaluate (27), which is more convenient and effective for investigating rural elderly populations.

In summary, this article suggests that when providing elderly people with nutritional knowledge and other interventions, changes their psychological state should be considered in addition to the sociodemographic differences in the elderly population. The local authorities can strengthen the promotion of nutritional literacy, enhance the elderly’s confidence in obtaining nutrition, and comprehensively improve the nutritional literacy level of the elderly population.

## 5 Conclusion

When conducting a nutritional literacy screening among rural elderly people, attention should be paid to the baseline characteristics of the elderly population: older age, elderly women, divorced or widowed, lower level of education, lower monthly living expenses, dental problems, and elderly people with chronic diseases tend to have lower nutritional literacy scores. Elderly people with low self-efficacy show a decline in nutritional literacy as they age, and those with multiple chronic diseases and low self-efficacy have lower nutritional literacy. Therefore, effective intervention measures should be taken for these key groups to promote a widespread improvement in nutritional literacy among the elderly population in rural western China.

## 6 Limitations

This study is the first to examine the nutritional status of elderly people in rural areas of northern Sichuan, and the sample size is limited based on the local rural elderly population. However, according to Kendall’s sample calculation method (50), the sample size of 264 cases meets the requirements by taking 5–10 times the number of entries in the most entries of the scale and considering a 20% sample loss rate. Furthermore, it is well known that people from different regions and cultural backgrounds have different health needs, which can lead to differences in nutritional status. The scale used in this study by Professor Zhang was selected to minimize the bias caused by geographic and cultural differences. Secondly, dietary habits and lifestyle are the basis for the development of nutritional status, and their nutritional status will ultimately move toward a direction favorable to chronic diseases. Since this study is a cross-sectional survey, it did not investigate the pre-nutrient status and course of chronic diseases of elderly people with these diseases, so more longitudinal studies are needed to explore the effect of chronic diseases on nutrient status. Finally, this study focused on rural elderly people in northern Sichuan, and the results cannot be generalized to other regions. Therefore, further research should expand the study population to comprehensively promote the improvement of nutritional status among rural elderly people.

## Data availability statement

The raw data supporting the conclusions of this article will be made available by the authors, without undue reservation.

## Ethics statement

The studies involving humans were approved by the Ethics Committee of Jinzhou Medical University (NO: JZMULL2022108). The studies were conducted in accordance with the local legislation and institutional requirements. The participants provided their written informed consent to participate in this study.

## Author contributions

SL: Conceptualization, Data curation, Investigation, Methodology, Project administration, Software, Writing – original draft. XF: Data curation, Supervision, Writing – review & editing. LJ: Investigation, Methodology, Writing – review & editing. TL: Formal analysis, Supervision, Writing – review & editing.

## References

[1] Acar TekNKaraçil-ErmumcuM. Determinants of health related quality of life in home dwelling elderly population: appetite and nutritional status. J Nutr Health Aging. (2018) 22:996–1002. doi: 10.1007/s12603-018-1066-9, PMID: 30272105

[2] Aihemaitijiang S Ye C Halimulati M Huang X Wang R Zhang Z. Development and validation of nutrition literacy questionnaire for the Chinese elderly. Nutrients. (2022) 14:1005. doi: 10.3390/nu14051005, PMID: 35267979 PMC8912634

[3] Fang EF Xie C Schenkel JA Wu C Long Q Cui H. A research agenda for ageing in China in the 21st century (2nd edition): focusing on basic and translational research, Long-term care, policy and social networks. Ageing Res Rev. (2020) 64:101174. doi: 10.1016/j.arr.2020.101174, PMID: 32971255 PMC7505078

[4] Sun H Li X Li W Feng J. Differences and influencing factors of relative poverty of urban and rural residents in China based on the survey of 31 provinces and cities. Int J Environ Res Public Health. (2022) 19:9015. doi: 10.3390/ijerph19159015, PMID: 35897386 PMC9332708

[5] Krause C Sommerhalder K Beer-Borst S Abel T. Just a subtle difference? Findings from a systematic review on definitions of nutrition literacy and food literacy. Health Promot Int. (2018) 33:378–89. doi: 10.1093/heapro/daw084, PMID: 27803197 PMC6005107

[6] Carbone ET Zoellner JM. Nutrition and health literacy: a systematic review to inform nutrition research and practice. J Acad Nutr Diet. (2012) 112:254–65. doi: 10.1016/j.jada.2011.08.042, PMID: 22732460

[7] Krabbenborg I de Roos N van der Grinten P Nap A. Diet quality and perceived effects of dietary changes in Dutch endometriosis patients: an observational study. Reprod Biomed Online. (2021) 43:952–61. doi: 10.1016/j.rbmo.2021.07.011, PMID: 34493462

[8] Shao JH Chen SH. Development and evaluation of a dietary self-management Programme for older adults with low literacy and heart disease: pilot study of feasibility and acceptability. J Adv Nurs. (2016) 72:3015–9. doi: 10.1111/jan.13075, PMID: 27434333

[9] Shin NM Choi J Cho I Park BJ. Self-management program for heart healthy behavior among middle- and old-aged Korean women at risk for metabolic syndrome. J Cardiovasc Nurs. (2017) 32:E8–e16. doi: 10.1097/jcn.0000000000000406, PMID: 28306702

[10] Donnelly HR Collins CE Haslam R White D Tehan PE. Perceptions of diet quality, advice, and dietary interventions in individuals with diabetes-related foot ulceration; a qualitative research study. Nutrients. (2022) 14:2457. doi: 10.3390/nu14122457, PMID: 35745190 PMC9228166

[11] Shao JH Chen SH. Who did it better? Gender differences in effects of a dietary self-management intervention for older community-dwelling adults. J Women Aging. (2021) 33:473–86. doi: 10.1080/08952841.2019.1707152, PMID: 31880992

[12] Aihara Y Minai J. Barriers and catalysts of nutrition literacy among elderly Japanese people. Health Promot Int. (2011) 26:421–31. doi: 10.1093/heapro/dar005 21307024

[13] JauhiainenLMännistöSYlöstaloPVehkalahtiMNordbladATurunenAW. Food consumption and nutrient intake in relation to denture use in 55- to 84-year-old men and women-results of a population based survey. J Nutr Health Aging. (2017) 21:492–500. doi: 10.1007/s12603-016-0793-z, PMID: 28448078

[14] Hajek A König HH. The role of optimism, self-esteem, and self-efficacy in moderating the relation between health comparisons and subjective well-being: results of a nationally representative longitudinal study among older adults. Br J Health Psychol. (2019) 24:547–70. doi: 10.1111/bjhp.12367 30927338

[15] Lee LO Grodstein F Trudel-Fitzgerald C James P Okuzono SS Koga HK. Optimism, daily stressors, and emotional well-being over two decades in a cohort of aging men. J Gerontol B Psychol Sci Soc Sci. (2022) 77:1373–83. doi: 10.1093/geronb/gbac025, PMID: 35255123 PMC9371455

[16] Whitehall L Rush R Górska S Forsyth K. The general self-efficacy of older adults receiving care: a systematic review and Meta-analysis. The Gerontologist. (2021) 61:e302–17. doi: 10.1093/geront/gnaa036, PMID: 32373938 PMC8361502

[17] Cybulski M Cybulski L Krajewska-Kulak E Cwalina U. The level of emotion control, anxiety, and self-efficacy in the elderly in Bialystok, Poland. Clin Intervent Aging. (2017) 12:305–14. doi: 10.2147/cia.S128717, PMID: 28223788 PMC5308481

[18] Su H Zhou Y Sun Y Cai Y. The relationship between depression and subjective cognitive decline in older adults of China: the mediating role of general self-efficacy. Psychol Health Med. (2022) 28:1057–67. doi: 10.1080/13548506.2022.2125165, PMID: 36165717

[19] Tielemans NS Schepers VP Visser-Meily JM Post MW van Heugten CM. Associations of proactive coping and self-efficacy with psychosocial outcomes in individuals after stroke. Arch Phys Med Rehabil. (2015) 96:1484–91. doi: 10.1016/j.apmr.2015.04.009, PMID: 25921978

[20] Berens EM Pelikan JM Schaeffer D. The effect of self-efficacy on health literacy in the German population. Health Promot Int. (2022) 37:daab085. doi: 10.1093/heapro/daab085, PMID: 34115848

[21] Young MD Plotnikoff RC Collins CE Callister R Morgan PJ. Social cognitive theory and physical activity: a systematic review and Meta-analysis. Obes Rev. (2014) 15:983–95. doi: 10.1111/obr.12225 25428600

[22] Zhang Y Zhang Z Xu M Aihemaitijiang S Ye C Zhu W. Development and validation of a food and nutrition literacy questionnaire for Chinese adults. Nutrients. (2022) 14:1933. doi: 10.3390/nu14091933, PMID: 35565900 PMC9104569

[23] Toniazzo MP Amorim PS Muniz F Weidlich P. Relationship of nutritional status and Oral health in elderly: systematic review with Meta-analysis. Clin Nutr. (2018) 37:824–30. doi: 10.1016/j.clnu.2017.03.014, PMID: 28392164

[24] Richard EL Laughlin GA Kritz-Silverstein D Reas ET Barrett-Connor E McEvoy LK. Dietary patterns and cognitive function among older community-dwelling adults. Nutrients. (2018) 10:1088. doi: 10.3390/nu10081088, PMID: 30110945 PMC6116163

[25] Bandura A. Self-efficacy: toward a unifying theory of behavioral change. Psychol Rev. (1977) 84:191–215. doi: 10.1037//0033-295x.84.2.191 847061

[26] Schwarzer R Jerusalem M. Measures in Health Psychology: A Users Portfolio. (1995), PMID: 29584644

[27] Leung DY Leung AY. Factor structure and gender invariance of the Chinese general self-efficacy scale among soon-to-be-aged adults. J Adv Nurs. (2011) 67:1383–92. doi: 10.1111/j.1365-2648.2010.05529.x, PMID: 21129011

[28] Gibbs H Chapman-Novakofski K. Establishing content validity for the nutrition literacy assessment instrument. Prev Chronic Dis. (2013) 10:E109. doi: 10.5888/pcd10.120267 23823698 PMC3702232

[29] Doustmohammadian A Keshavarz Mohammadi N Omidvar N Amini M Abdollahi M Eini-Zinab H. Food and nutrition literacy (Fnlit) and its predictors in primary schoolchildren in Iran. Health Promot Int. (2019) 34:1002–13. doi: 10.1093/heapro/day050, PMID: 30101341

[30] Ayer Ç Ergin A. Status of nutritional literacy in adolescents in the semi-rural area in Turkey and related factors. Public Health Nutr. (2021) 24:3870–8. doi: 10.1017/s1368980021002366, PMID: 34047263 PMC8369453

[31] Hayes AF Matthes J. Computational procedures for probing interactions in Ols and logistic regression: Spss and Sas implementations. Behav Res Methods. (2009) 41:924–36. doi: 10.3758/brm.41.3.924, PMID: 19587209

[32] Haugland T Wahl AK Hofoss D DeVon HA. Association between general self-efficacy, social support, Cancer-related stress and physical health-related quality of life: a path model study in patients with neuroendocrine tumors. Health Qual Life Outcomes. (2016) 14:11. doi: 10.1186/s12955-016-0413-y, PMID: 26787226 PMC4717553

[33] Whitelock E Ensaff H. On your own: older Adults’ food choice and dietary habits. Nutrients. (2018) 10:413. doi: 10.3390/nu10040413, PMID: 29584644 PMC5946198

[34] Liu A Ding C Qiu Y Yuan F Wang Q Hong H. Age distribution of nutrition and health knowledge among Chinese adults in 2021. J Hyg Res. (2022) 51:876–80. doi: 10.19813/j.cnki.weishengyanjiu.2022.06.004 36539861

[35] Liu X Yu S Mao Z Li Y Zhang H Yang K. Dyslipidemia prevalence, awareness, treatment, control, and risk factors in Chinese rural population: the Henan rural cohort study. Lipids Health Dis. (2018) 17:119. doi: 10.1186/s12944-018-0768-7, PMID: 29788966 PMC5964901

[36] Murakami K Shinozaki N Livingstone MBE Yuan X Tajima R Matsumoto M. Associations of food choice values and food literacy with overall diet quality: a Nationwide cross-sectional study in Japanese adults. Br J Nutr. (2023) 130:1795–805. doi: 10.1017/s000711452300082x 37017207 PMC10587391

[37] Shah SJ Fang MC Wannier SR Steinman MA Covinsky KE. Association of Social Support with functional outcomes in older adults who live alone. JAMA Intern Med. (2022) 182:26–32. doi: 10.1001/jamainternmed.2021.6588, PMID: 34779818 PMC8593829

[38] Wang S Yang Y Hu R Long H Wang N Wang Q. Trends and associated factors of dietary knowledge among Chinese older residents: results from the China health and nutrition survey 2004-2015. Int J Environ Res Public Health. (2020) 17:8029. doi: 10.3390/ijerph17218029, PMID: 33142725 PMC7662652

[39] Schlüter K Vamos S Wacker C Welter VDE. A conceptual model map on health and nutrition behavior (CMM^HB/NB^). Int J Environ Res Public Health. (2020) 17:7829. doi: 10.3390/ijerph17217829, PMID: 33114729 PMC7663323

[40] Lobo E Tamayo M Sanclemente T. Nutrition literacy and healthy diet: findings from the validation of a short seniors-oriented screening tool, the Spanish myths-Nl. Int J Environ Res Public Health. (2021) 18:12107. doi: 10.3390/ijerph182212107, PMID: 34831865 PMC8624156

[41] Zhou Y Guo Y Liu Y. Health, income and poverty: evidence from china’s rural household survey. International journal for equity in health. (2020) 19(1):36. Epub 2020/03/18. doi: 10.1186/s12939-020-1121-0 PMID: 32178686 PMC7076955

[42] Bessems K Linssen E Lomme M van Assema P. The effectiveness of the good affordable food intervention for adults with low socioeconomic status and small incomes. Int J Environ Res Public Health. (2020) 17:2535. doi: 10.3390/ijerph17072535, PMID: 32272792 PMC7178221

[43] Gao YB Hu T Zhou XD Shao R Cheng R Wang GS. Dental caries in Chinese elderly people: findings from the 4th National Oral Health Survey. Chinese J Dent Res. (2018) 21:213–20. doi: 10.3290/j.cjdr.a41077, PMID: 30255172

[44] Hu FB. Dietary pattern analysis: a new direction in nutritional epidemiology. Curr Opin Lipidol. (2002) 13:3–9. doi: 10.1097/00041433-200202000-00002 11790957

[45] Rivera RL Adams M Dawkins E Carter A Zhang X Tu W. Delivering food resources and kitchen skills (forks) to adults with food insecurity and hypertension: a pilot study. Nutrients. (2023) 15:1452. doi: 10.3390/nu15061452, PMID: 36986184 PMC10051267

[46] Guntzviller LM King AJ Jensen JD Davis LA. Self-efficacy, health literacy, and nutrition and exercise behaviors in a low-income, Hispanic population. J Immigr Minor Health. (2017) 19:489–93. doi: 10.1007/s10903-016-0384-4 26979167

[47] Guttersrud Ø Petterson KS. Young Adolescents’ engagement in dietary behaviour - the impact of gender, socio-economic status, self-efficacy and scientific literacy. Methodological aspects of constructing measures in nutrition literacy research using the Rasch model. Public Health Nutr. (2015) 18:2565–74. doi: 10.1017/s1368980014003152, PMID: 25634262 PMC10271695

[48] Aierken A Ding X Pan Y Chen Y Li Y. Association between dependency on community resources and social support among elderly people living in rural areas in China: a cross-sectional study. BMC Geriatr. (2022) 22:589. doi: 10.1186/s12877-022-03247-5, PMID: 35842579 PMC9288718

[49] ChauPHNgaiHHLeungAYLiSFYeungLOTan-UnKC. Preference of food saltiness and willingness to consume low-sodium content food in a Chinese population. J Nutr Health Aging. (2017) 21:3–10. doi: 10.1007/s12603-016-0732-z, PMID: 27999843

[50] Preacher KJ Kelley K. Effect size measures for mediation models: quantitative strategies for communicating indirect effects. Psychol Methods. (2011) 16:93–115. doi: 10.1037/a0022658, PMID: 21500915

