# Supplementary material for: A cross-sectional study on the moderating effect of self-efficacy on the relationship between sociodemographic variables and nutrition literacy among older adults in rural areas of North Sichuan
Source: Front Nutr. 2024 Jan 8;10:1335008. doi: 10.3389/fnut.2023.1335008 (PMC10800664; doi:10.3389/fnut.2023.1335008)
Supplement: Supplementary file 1 [file Table_1.pdf]

Table S1 Comparison of pairwise demographic variables within the group

	Characteristics	n (%)	NLQ-E(median)	P	Pairwise comparison
Age	①60-69	115（43.6%）	80（77，97）	<0.001	①>②>③
	②70-79	109（41.3%）	77（66，80）		
	③≥80	40（15.2%）	62（40，72）		
Marriage status	①Married	200（75.8%）	78（71，88）	<0.05	①>②
	②Divorced/Widowhood	63（23.9%）	72（61，80）		
Education level	①Illiteracy	97（36.7%）	66（51，77）	<0.05	③>②>①
	②Primary school diploma	94（35.6%）	79（73，82）		
	③Junior high school diploma	70（26.5%）	88（80，98）		
living expenses CNY/month	①≤500	101（38.3%）	68（53，78）	<0.001	③>②>①
	②501-999	112（42.4%）	78（73，87）		
	③1000-1999	46（17.4%）	97（81，100）		
Tooth condition	①false tooth	100(37.9%)	73（66，78）	<0.001	③>①<br>③>②
	②Damaged teeth without dentures	90(34.1%)	78（66，80）		
	③Intact teeth	71(26.9%)	84（77，97）		
Chronic diseases	①0	117(44.3%)	80（77，97）	<0.001	①>②<br>①>③
	②1	113(42.8%)	72（66，80）		
	③2	32(12.1%)	71（52，80）		
